# A MS‐lesion pattern discrimination plot based on geostatistics

**DOI:** 10.1002/brb3.430

**Published:** 2016-01-30

**Authors:** Robert Marschallinger, Paul Schmidt, Peter Hofmann, Claus Zimmer, Peter M. Atkinson, Johann Sellner, Eugen Trinka, Mark Mühlau

**Affiliations:** ^1^ Interfaculty Department of Geoinformatics Z_GISUniv. SalzburgSchillerstr. 305020SalzburgAustria; ^2^ Department of NeurologyChristian Doppler Medical CentreParacelsus Medical UniversityIgnaz Harrer‐Straße 795020SalzburgAustria; ^3^ Department of NeurologyKlinikum rechts der IsarTechnische Universität MünchenMunichGermany; ^4^ TUM–Neuroimaging CenterKlinikum rechts der IsarTechnische Universität MünchenMunichGermany; ^5^ Department of StatisticsLudwig‐Maximilians‐University MünchenMunichGermany; ^6^ Department of NeuroradiologyKlinikum rechts der IsarTechnische Universität MünchenMunichGermany; ^7^ Faculty of Science and TechnologyLancaster UniversityEngineering BuildingLancasterLA1 4YRUK; ^8^ Faculty of GeosciencesUniversity of UtrechtHeidelberglaan23584 CSUtrechtThe Netherlands; ^9^ School of Geography, Archaeology and PalaeoecologyQueen's University BelfastBelfastBT7 1NNNorthern IrelandUK; ^10^ Munich Cluster for Systems Neurology (SyNergy)MunichGermany

**Keywords:** Discrimination, geostatistics, lesion, Multiple Sclerosis, pattern

## Abstract

**Introduction:**

A geostatistical approach to characterize MS‐lesion patterns based on their geometrical properties is presented.

**Methods:**

A dataset of 259 binary MS‐lesion masks in MNI space was subjected to directional variography. A model function was fit to express the observed spatial variability in *x*,* y*,* z* directions by the geostatistical parameters Range and Sill.

**Results:**

Parameters Range and Sill correlate with MS‐lesion pattern surface complexity and total lesion volume. A scatter plot of ln(Range) versus ln(Sill), classified by pattern anisotropy, enables a consistent and clearly arranged presentation of MS‐lesion patterns based on geometry: the so‐called MS‐Lesion Pattern Discrimination Plot.

**Conclusions:**

The geostatistical approach and the graphical representation of results are considered efficient exploratory data analysis tools for cross‐sectional, follow‐up, and medication impact analysis.

## Introduction

Multiple sclerosis (MS) is an inflammatory demyelinating disease of the central nervous system with neurodegenerative processes in the later course. It affects over 2.5 million people worldwide and is the leading nontraumatic cause of serious neurologic disability in young adults. MS is characterized by unpredictable episodes of clinical relapses and remissions followed by continuous progression of disability over time (secondary progressive MS) in most instances. The course of MS is highly variable – from benign to disastrous (Compston and Coles 2008). While some patients may acquire severe and irreversible disability within a few years, others may run a benign course with little or no disability even after decades. The hallmark of MS is sclerotic lesions within cerebral white matter, which are hyperintense on T2‐weighted brain MRI sequences. These lesions present rather heterogeneously across patients not only with regard to the number and overall volume but also with regard to spatial pattern, predilection sites, and shape of single lesions (Filippi and Rocca 2011). Researching the geometrical configurations of white matter MS‐lesions from MRI investigation is considered an opportunity for greater understanding of the relationship between MS clinics and neuroradiological findings (Pham et al. 2010; Marschallinger et al. 2014; Taschler et al. 2014). Until now, the heterogeneity of MRI findings could not be related fully to the heterogeneity of the disease course. This may be achieved by the application of mathematical tools, which are not well established in neuroimaging. Here, we aim to characterize white matter lesions in MS using measures from geostatistics. With the advent of brain geometry normalization (Penny et al. 2007) and automatic MS‐lesion segmentation (Garcia‐Lorenzo et al. 2012), large numbers of classified images can be made available for continued evaluation.

For this study, we define a MS‐lesion pattern as the ensemble of MS‐lesions identified in a specific MRI examination of a single patient. In a pilot evaluation of the approach followed here (Marschallinger et al. 2014), a small yet representative dataset of three synthetic and three manually segmented real‐world MS‐lesion patterns was used to show the potential of geostatistics to yield key geometrical information on MS‐lesion patterns. This study applies the geostatistical approach to 259 automatically segmented binary MS‐lesion patterns that are representative of probable MS‐lesion pattern geometries.

## Materials and Methods

### The dataset

We analyzed lesion maps of 259 patients. The median score on the Expanded Disability Status Scale (EDSS) was 1.3 (standard deviation, 1.0; median, 1.5; range, 0–6.0), median age was 37 years (standard deviation 10; median, 36; range 19–70), and mean disease duration was 3.1 years (standard deviation, 2.3; median, 2.7; range, 0.1–10). Forty‐four patients had experienced one (clinically isolated syndrome) and 215 more than one demyelinating attack (relapsing‐remitting MS). The female/male ratio was 175/84.

**Figure 1:**
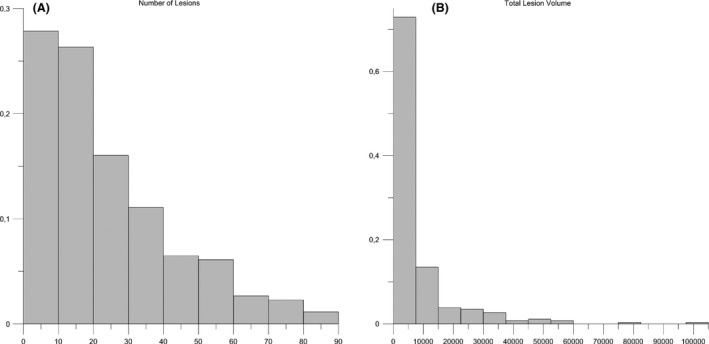
(A) Number of lesions (min = 1, max = 86, mode = 10). (B) Total lesion volume mm^3^ (min = 54, max = 102,583, mode = class 0–1000).

The dataset consists of 259 binary MS‐lesion patterns projected to the MNI space. Dimensions of the voxel arrays are (x*y*z) 121*145*121 voxels, with 1.5*1.5*1.5 mm^3^ per voxel, with the MS‐lesion voxels assigned to gray level 1, and the remaining (void) voxels to gray level 0. For the remainder of this study, we refer to this binary, normalized dataset as the “MS‐259 dataset”. The histograms in Figure 1(A and B) show the number of lesions and total lesion volume across this MS‐259 dataset. Both distributions are approximately log‐normally skewed. The number of lesions in each image pattern varies between 1 and 86 lesions, with a mode of 12 lesions. The total lesion volume varies between 54 and 102,583 mm^3^, with the most frequent class between 0 and 1000 mm^3^.

### Work flow and software

#### LST: MS‐lesion segmentation

Lesions were segmented by an automated tool, the lesion segmentation tool (LST), which is freely available (www.applied-statistics.de/lst.html). It is an extension of the voxel‐based morphometry toolbox (www.neuro.uni-jena.de/vbm8/) of the software package Statistical Parametric Mapping (SPM) 8 (www.fil.ion.ucl.ac.uk/spm/). The algorithm requires a three‐dimensional (3D) gradient echo (GRE) T1‐weighted and a FLAIR image at 3 Tesla (T). It determines the three tissue classes of gray matter (GM) and WM as well as cerebrospinal fluid (CSF) from the T1‐weighted image, and, then, the FLAIR intensity distribution of each tissue class in order to detect outliers, which are interpreted as lesion beliefs. Next, a conservative lesion belief is expanded toward a liberal lesion belief. To this end, neighboring voxels are analyzed and assigned to lesions under certain conditions. This is done iteratively until no further voxels are assigned to lesions. Here, the likelihood of belonging to WM or GM is weighted against the likelihood of belonging to lesions (Schmidt et al. 2012). Finally, 3D binary lesion maps in MNI space are generated, which were used here.

**Figure 2:**
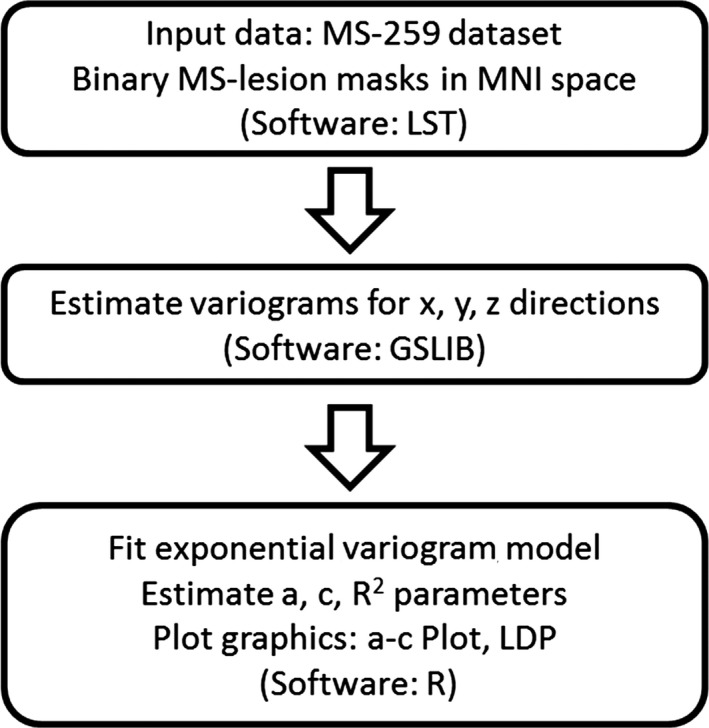
Workflow for characterizing MS‐lesion patterns by means of geostatistics. See text for details.

The workflow followed in this study is depicted in Figure 2: Per pattern (MS‐lesion mask), three directional empirical variograms are estimated at orthogonal orientations in 3D. Per empirical variogram, a variogram model function is fitted that provides a summary description of the pattern by means of two parameters: Range (*a*) and Sill (*c*). Parameters *a* and *c* are expressed in classified scatterplots to provide a straightforward presentation of the geometrical summary characteristics of MS‐lesion patterns.

#### Empirical variograms

Geostatistics provides algorithms for characterizing, modeling, and simulating multidimensional data in a variety of disciplines (Conan et al. 1992; Christakos 2000; Kourgli et al. 2004; Blewett and Kildluff 2006; Caers 2010). The variogram, a measure of spatial correlation, is a central tool in geostatistics and can be used for exploratory data analysis (EDA) (Gringarten and Deutsch 1999). Applied to binary MS‐lesion patterns from MRI, variography enables characterization and quantification of the geometrical properties of MS‐lesion patterns (Marschallinger et al. 2009). When MS‐lesion patterns are normalized to MNI space, variography enables single patient follow‐up analysis, and intra or intergroup analysis (Marschallinger et al. 2014). The empirical variogram *γ*(**h**) is calculated using (eq. (1)):

(1) $$\gamma \left(h\right)=\frac{1}{2n(h)}\cdot \sum_{i=1}^{n}{\left((z\left(x_{i}\right)-z\left(x_{i}+h\right)\right)}^{2}$$

*z*(**x**) value of variable at some 3D location **x**, here: voxel with *z * =  binary variable (0 or 1); **h** lag vector of separation between observed data (units: mm); *n*(**h**) number of data pairs [*z*(**x**), *z*(**x**+**h**)] at lag **h;** *γ*(**h**) empirical variogram value for lag **h**.

**Figure 3:**
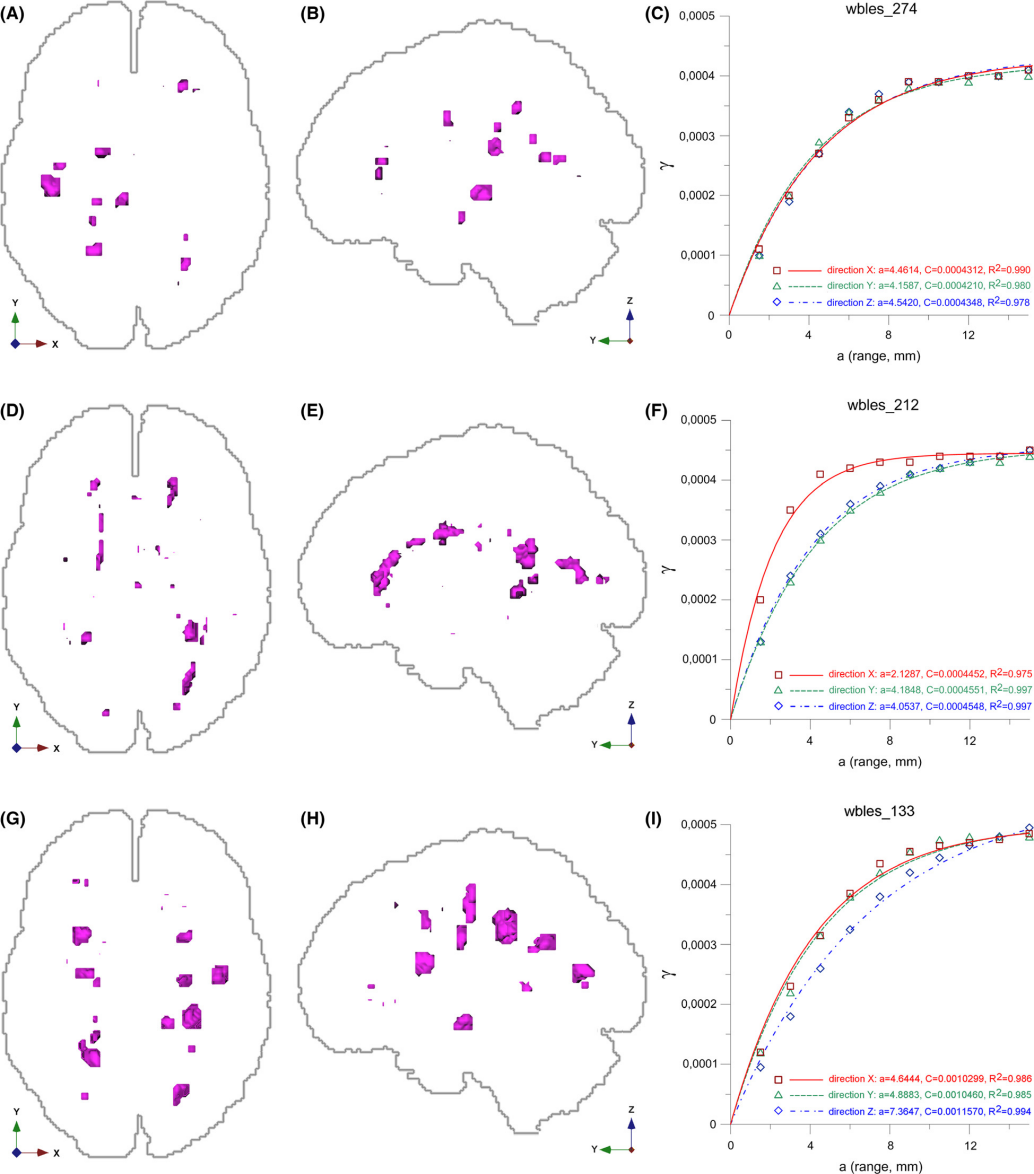
Relation of MS‐lesion pattern geometry, empirical variograms, and fitted variogram models and their estimated parameters. Projections of three MS‐lesion patterns (magenta) to (left image) axial and (middle image) sagittal planes, white matter outlines (light gray), and axis tripod (directions) for reference. (A, B) Case wbles_274, pattern with dominantly geometrically isotropic MS‐lesions, total lesion load = 2666 mm^3^. (D, E) Case wbles_212, pattern with dominantly geometrically anisotropic MS‐lesions, total lesion load = 2943 mm^3^. (G, H) case wbles_133, pattern with dominantly geometrically anisotropic MS‐lesions, total lesion load = 6571 mm^3^. (C, F, I) Associated directional empirical variograms, fitted exponential variogram models, estimated *a* and *c* parameters and quality of model fitting (*R* ^2^). Color coding of directional empirical variograms and variogram models: Red *X*, green: *Y*, blue: *Z*. See text for details.

The *γ*(**h**) of a binary MS‐lesion pattern is estimated by comparing the binary values (0 or 1) of all voxel pairs within a specified lag **h** according to equation (1). Calculating *γ*(**h**) for increasing lag distances ¦**h**¦, the empirical variogram plot (“the variogram”) is derived (Fig. 3C, F, I). Computing variograms for specific lag orientations yields directional variograms that quantify spatial anisotropies in the data. Variograms of MS‐lesion patterns generally start with small values of *γ* at small ¦**h**¦, reflecting the large correlation of adjacent voxel pairs (neighboring voxels tend to have the same binary value). After an initial increase with lag away from the origin, with further increases in ¦**h**¦ the correlation decreases, and eventually the variogram begins to level off. As a rule of thumb, the flatter the variogram near the origin, the more pronounced is the spatial correlation (i.e., the larger the lesions will be). As pointed out in (Marschallinger et al. 2014), variograms of binary MS‐lesion patterns should be confined to distances from 0 to 15 mm, because this area holds most of the relevant correlation information and a variogram model can be fitted straightforwardly; a more detailed introduction to using variography with MRI datasets is given there.

Since the LST algorithm provides binary MS‐lesion patterns in MNI space, LST results can be interpreted directly using variography. For each member of the MS‐259 dataset directional empirical variograms were estimated in the three main orthogonal orientations *X*,* Y*,* Z* (dextral‐sinistral, caudal‐rostral, dorsal‐ventral orientations), within distances between 0 and 15 mm.

Figure 3 shows the sensitivity of the directional variograms (Deutsch and Journel 1997) to MS‐lesion pattern geometry. It contrasts three MS‐lesions patterns and the associated variograms. Case Wbles_274 has dominantly isotropic (spherical) lesions. Accordingly the variograms for *X*,* Y*,* Z* directions show approximately the same shape, indicating similar spatial correlation in all directions. Most MS‐lesions in wbles_212 are anisotropic; they are stretched in the *Y* and *Z* directions. Here, the variograms for the *Y* and *Z* directions exhibit a shallower slope near the origin than for the *X* direction, indicating greater spatial continuity in the *Y* and *Z* than *X* directions. In wbles_133 the majority of the MS‐lesions are stretched in the *Z* direction; as a consequence, the variogram for the *Z* direction has the shallowest slope near the origin.

#### Variogram models

Empirical variograms are graphical representations of spatial correlation, primarily intended for visual analysis. Several permissible variogram model functions exist for quantification (Cressie 1993): After being fitted to an empirical variogram, these model functions express a variogram's shape by the model type (e.g., exponential, spherical) and commonly two parameters: the variogram range *a**,*** and the variogram sill *c*. Among the available and permissible variogram model functions, the exponential variogram model (eq. (2)) was found to be the most suitable for quantifying the MS‐lesion patterns (Marschallinger et al. 2014).

(2) $$\gamma \left(h\right)=c\cdot \left(1-\frac{e^{\left(-3\cdot |h|\right)}}{a}\right)$$

*c* Sill; *a* Range; **h** lag vector of separation; *γ*(**h**) model variogram value for lag **h**.

Figure 3(C, F, I) illustrate the process of variogram model fitting. They combine directional empirical variograms (symbols: red square = *X*, green triangle = *Y*, blue diamond = *Z* direction), the fitted exponential variogram model functions (lines: red continuous = *X*, green dash = *Y*, blue dash‐dot = Z direction), the estimated *a* and *c* parameters and the goodness‐of‐fit (*R* ^2^). Model fitting and parameter estimation were computed with the software R (R Development Core Team, 2008): the range *a* is roughly the same in the *X*,* Y*,* Z* directions for wbles_274, indicating a nearly isotropic pattern. In contrast, for wbles_212 the range *a* in the *X* direction is just about half of *a* in the *Y* and *Z* directions, indicating greater spatial correlation in the *Y* and *Z* directions. This is confirmed by Figure 3(D and E) where the majority of the MS‐lesions are stretched in the *Y* and *Z* directions. The dominant stretching of MS‐lesions in the *Z* direction in pattern wbles_133 is expressed by a larger range in the *Z* direction. The sill *c* increases in the order wbles_212, wbles_274, wbles_133, but is similar per pattern.

**Figure 4:**
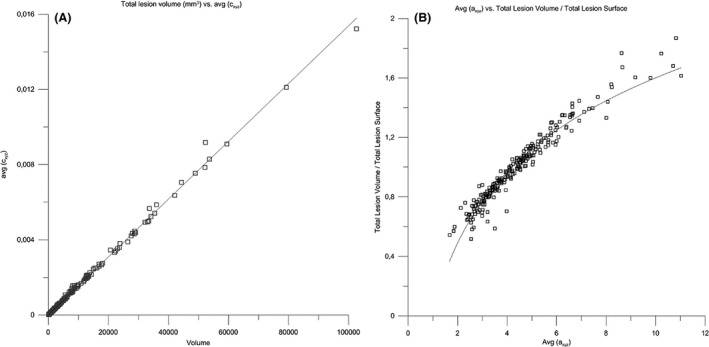
Correlations of geostatistical parameters *a* and *c* with total volume and total surface area of MS‐lesion patterns in the MS‐259 dataset. (A) Correlation of total lesion volume with *c* (*R* ^2^ = 0.997). (B) Correlation of (total lesion volume/total lesion surface) with *a* (*R* ^2^ = 0.941).

The variogram measures spatial continuity (or discontinuity), which in the case of 3D structures can be interpreted as surface complexity (Kourgli et al. 2004; Trevisani et al. 2009). The surface complexity of biological structures is often expressed as the ratio of surface area and volume (Schmidt‐Nielson 1984). To cross‐check the correlation between the *a* and *c* parameters and lesion pattern surface complexity, the total lesion volume (mm^3^), and total lesion surface area (mm^2^) were calculated for each pattern of the MS‐259 dataset. Correlating parameters *a* and *c* with total lesion surface area and total lesion volume (Vtot) reveals an almost perfect linear correlation (*R* ^2^ = 0.997) between *c* (sill) and Vtot. Furthermore, there is a significant log correlation (*R* ^2^ = 0.935) of *a* with the ratio of the total lesion volume/total lesion surface area. In other words: in binary MS‐lesion patterns, the variogram model sill *c* is a substitute for total lesion load (Fig. 4A) and the model range *a* is a proxy of MS‐lesion pattern surface complexity (Fig. 4B). The greater *c*, the greater is the total lesion volume. The greater *a*, the greater is the overall spatial correlation and the smoother (i.e., the less complex) is the pattern's surface.

#### *a*‐*c* plot

In geostatistics, the fitted variogram model range *a* and sill *c* are used to convey information for use in geostatistical operations such as spatial prediction and simulation (Isaaks and Srivastava 1989). In the current context, *a* and *c* are used to characterize MS‐lesion pattern geometry by three value pairs: *a*_*[X]*_ *,c*_*[X]*_ *; a*_*[Y]*_ *,c*_*[Y]*_ *; a*_*[Z]*_ *,c*_*[Z]*_ *; (*with *a*_*[x]*_ *,c*_*[X]*_ *; … values of a, c in direction x, etc.)*. When lesion patterns are geometrically normalized, their geometry can be conveniently portrayed and compared in a diagram of *a* versus *c* (*ac*‐plot (Marschallinger et al. 2014)).

**Figure 5:**
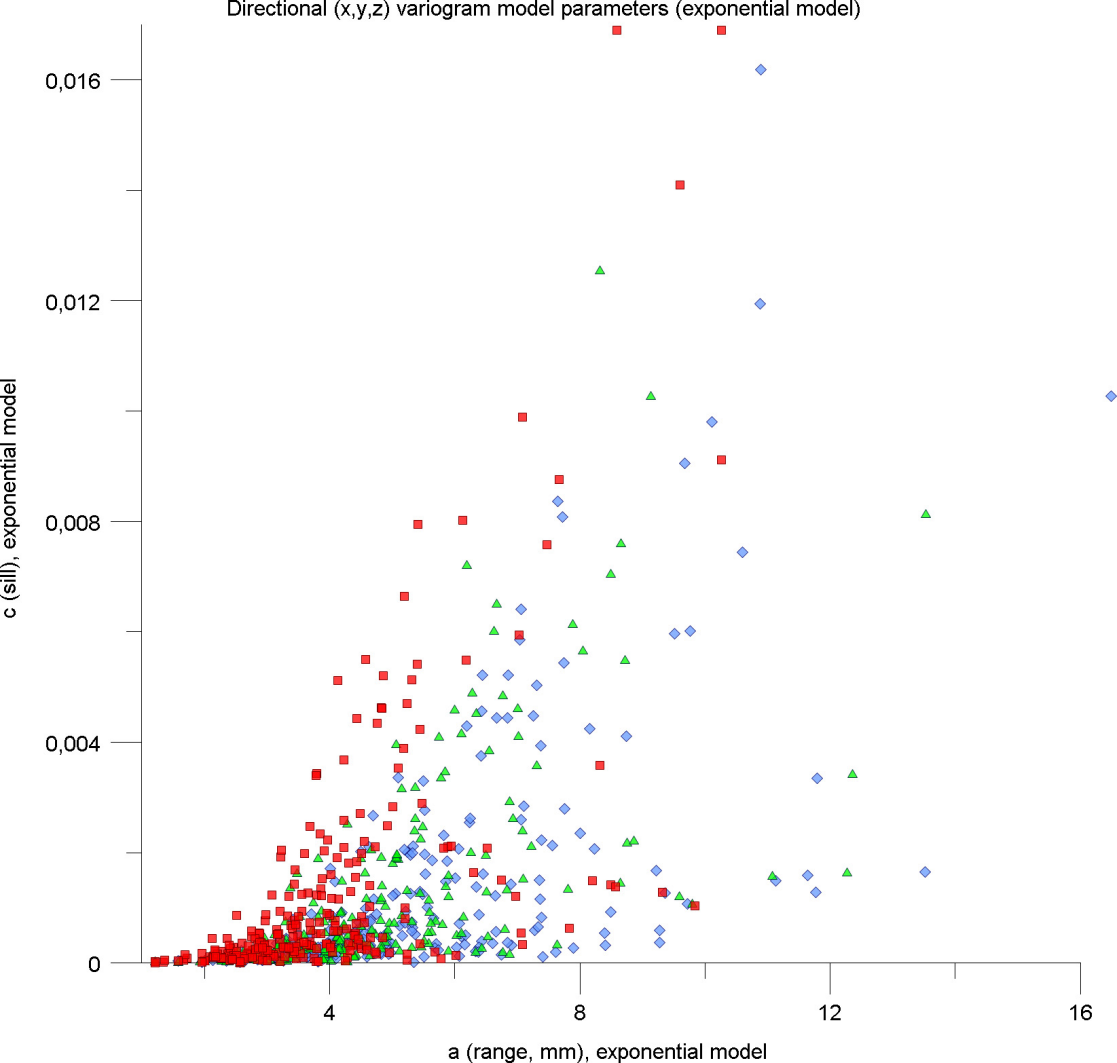
Scatterplot *a*_*[X]*_ *,c*_*[X]*_ *; a*_*[Y]*_ *,c*_*[Y]*_ *; a*_*[Z]*_ *,c*_*[Z]*_ for the MS‐259 dataset as a whole. Each of the 259 MS‐lesion patterns is represented by three symbols: *X*‐direction – red squares, *Y*‐direction – green triangles, *Z*‐direction – blue diamonds. See text for details.

**Figure 6:**
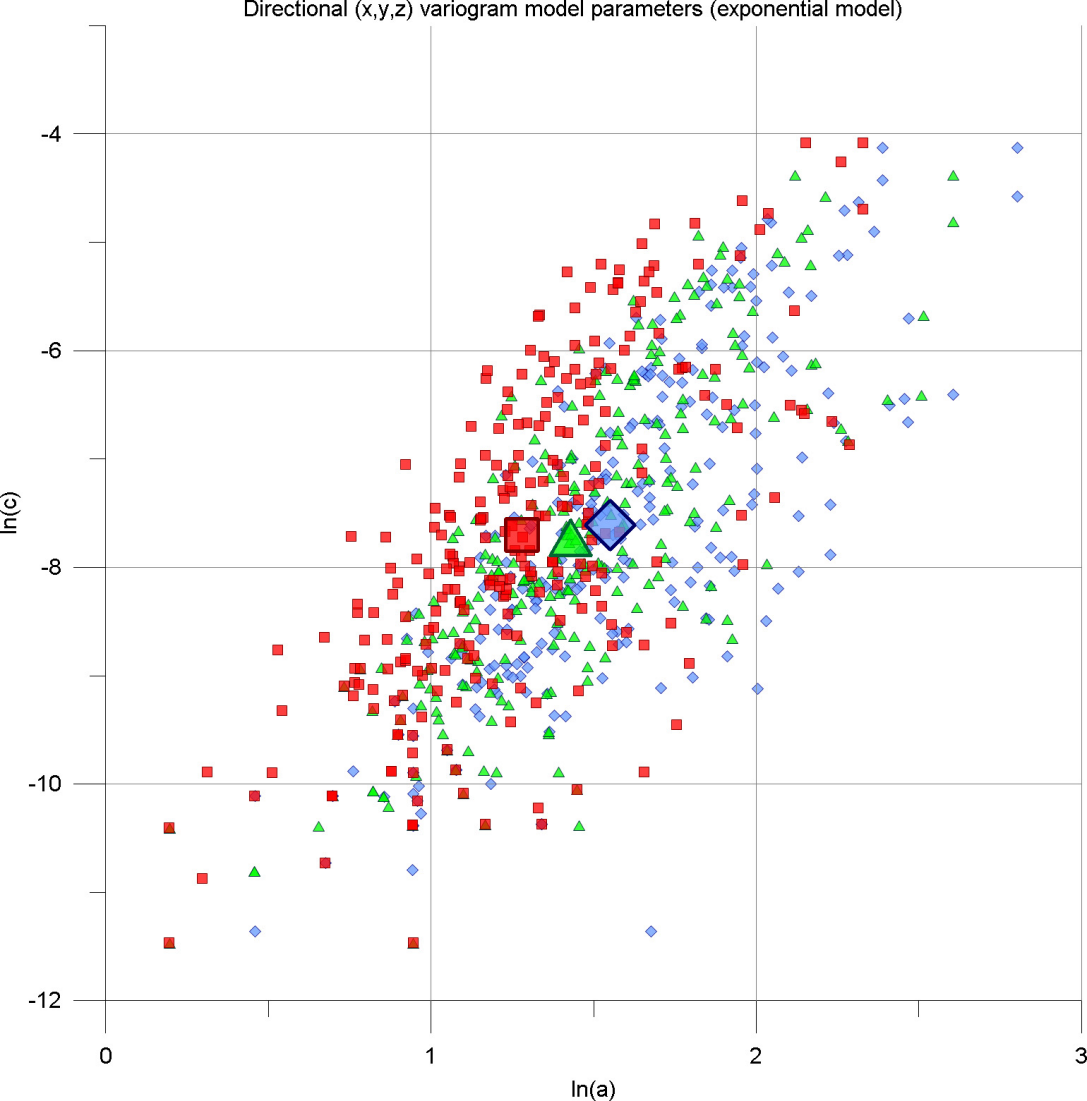
a‐c plot involving ln(*a*_[X,Y,Z]_) versus ln(*c*_[X,Y,Z]_) for the MS‐259 dataset. Directional components: *X* – red squares, *Y* – green triangles, *Z* – blue diamonds. Large symbols are the spatial means. See text for details.

Figure 5 is a plot of *a* (abscissa) versus *c* (ordinate) for the MS‐259 dataset. The plot shows dense clustering near the origin that obscures detail, and a possible bifurcation at medium to large *a‐c* values. To overcome the clustering, natural log scaling was applied to both the *a* and *c* axes in Figure 6.

Figure 6 provides a clearer synopsis: visually, the densely clustered points at small *a,c* values are stretched while large *a,c* values are compressed. For the MS‐259 dataset, ln(*a*_*[X,Y,Z]*_) is between 0.20 and 2.80, and ln(*c*_*[X,Y,Z]*_) is between −11.47 and −4.08. Since the MS‐259 dataset comprises a broad range of very mild to extremely severe cases, we consider that the majority of possible MS‐lesion patterns will plot within the axis limits of ln(*a*) = [0.3] and ln(*c*) = [−12,−3]. In Figure 6, the MS‐259 dataset forms a loose, elliptic cloud with the long axis running about diagonal. Within the cloud, the *X*,* Y,* and *Z* directional components also form elliptic, overlapping areas. The visually discernible shift towards larger ln(*a*), with *a*_*[X]*_ <*a*_*[Y]*_ <*a*_*[Z]*_ is confirmed by the respective mean centers (mean center definition see below). At the individual level, the vast majority of the MS‐259 dataset lesion patterns show varying *a*_[X,Y,Z],_ but similar *c*_[X,Y,Z]_ values (the so‐called “geometric anisotropy”).

**Figure 7:**
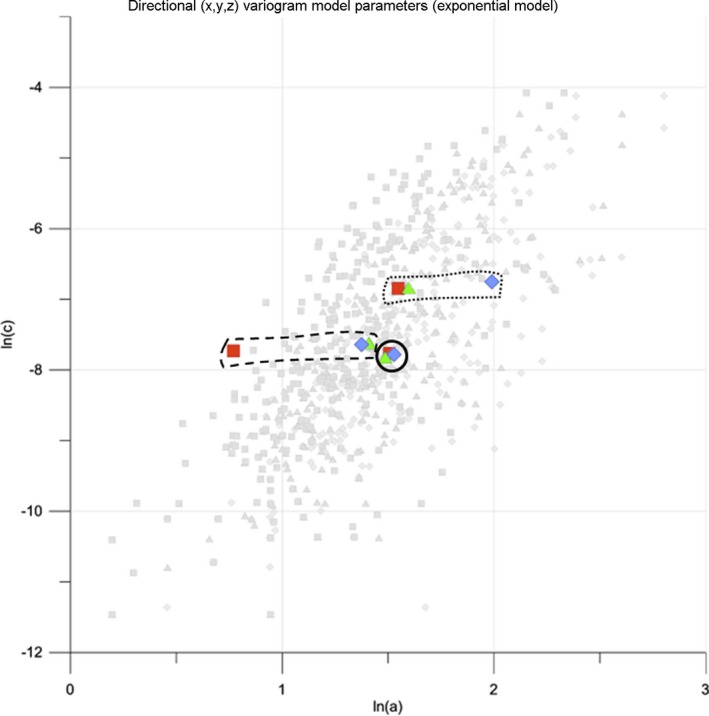
a‐c plot for distinguishing spatially isotropic lesion patterns (case wbles_274: continuous line) from spatially anisotropic lesion patterns (case wbles_212: dashed line, case wbles_133: dotted line). Symbols for directional components are the same as in Figure 6. Light‐gray backdrop is MS‐259 dataset for reference.

Figure 7 compares an isotropic and two anisotropic MS‐lesion patterns in the *a‐c* plot (patterns in Figure 3): the *X*,* Y*,* Z* symbols of the isotropic wbles_274 pattern plot closely together. The symbols of the anisotropic pattern wbles_212 clearly indicate a smaller *a* for *X* than for the *Y* and *Z* directions (lesion elongation in the *Y* and *Z* directions), whereas the anisotropy of wbles_133 is expressed by a larger *a* for *Z* than for the *X* and *Y* directions (lesion elongation in the *Z* direction). This is in accordance with the observed lesion pattern geometries in Figure 3. As such, the *a*‐*c* plot straightforwardly communicates geometric anisotropy of MS‐lesion patterns. While the geometrical characteristics of single patterns can be represented conveniently by separate *X*,* Y*,* Z* symbols per pattern, this can be confusing for larger datasets. When presenting many MS‐lesion patterns in the *a‐c* plot, it makes sense to identify each pattern with only one point and to express the magnitude of anisotropy by symbol classes. The mean center (eq. (3a), (3b)) is widespread for representing average location, in the current context:

(3a) $$\overset{¯}{a}=\frac{\sum_{i=1}^{n}a_{i}}{n}$$

(3b) $$\overset{¯}{c}=\frac{\sum_{i=1}^{n}c_{i}}{n}$$

$\overset{¯}{a}$ mean *a* (average geostatistical range); $\overset{¯}{c}$ mean *c* (average geostatistical sill); *n* number of data, here: 3 (*x*,* y*,* z*).

Analogous to the standard deviation in univariate statistics, the standard distance (“SD”, eq. (4)) indicates deviation from the spatial mean (De Smith et al. 2007). The more the SD deviates from 0 (the isotropic case), the more anisotropic a lesion pattern is. In the MS‐259 dataset, the SD in the *a*‐*c* plot varies between 0.001 and 0.473.

(4) $$\text{SD}=\sqrt{\frac{\sum_{i=1}^{n}{\left(a_{i}-\overset{¯}{a}\right)}^{2}}{n}+\frac{\sum_{i=1}^{n}{\left(c_{i}-\overset{¯}{c}\right)}^{2}}{n}}$$

$\overset{¯}{a}$ mean *a* (average geostatistical range); $\overset{¯}{c}$ mean *c* (average geostatistical sill); *n* number of data, here: 3 (*x*,* y*,* z*).

**Figure 8:**
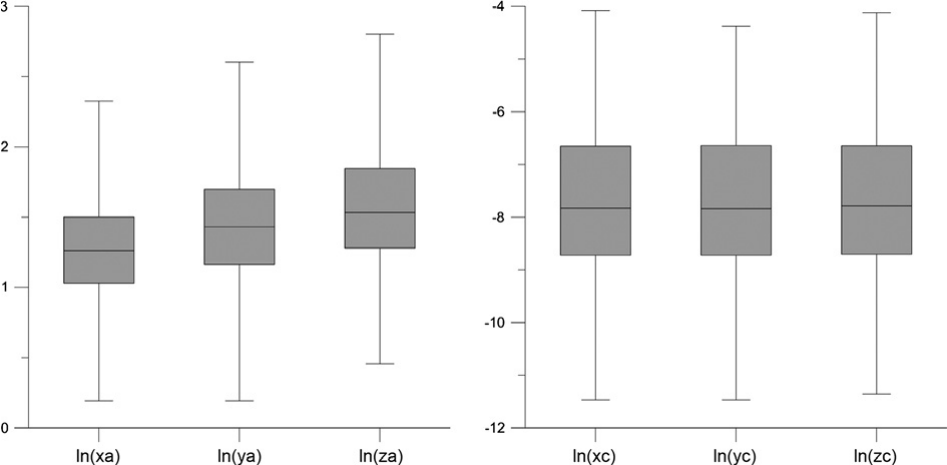
Box‐plots of Figure 6 marginal distributions: ln(xa), ln(ya), ln(za), ln(xc), ln(yc), ln(zc)). Box is bounded by first and third quartile, line in box is median. Whiskers indicate minimum and maximum. See text for details.

Mean center and standard distance are used here to express average location and spatial spread in *a‐c* space because both marginal distributions can be considered normal: both ln (*a*) and ln (*c*) of the a‐c plot are almost perfect normal distributions for all (*x*,* y*,* z*) directional variograms. This is confirmed by Figure 8 which gives the relevant box‐plots.

#### MS‐lesion pattern discrimination plot

**Figure 9:**
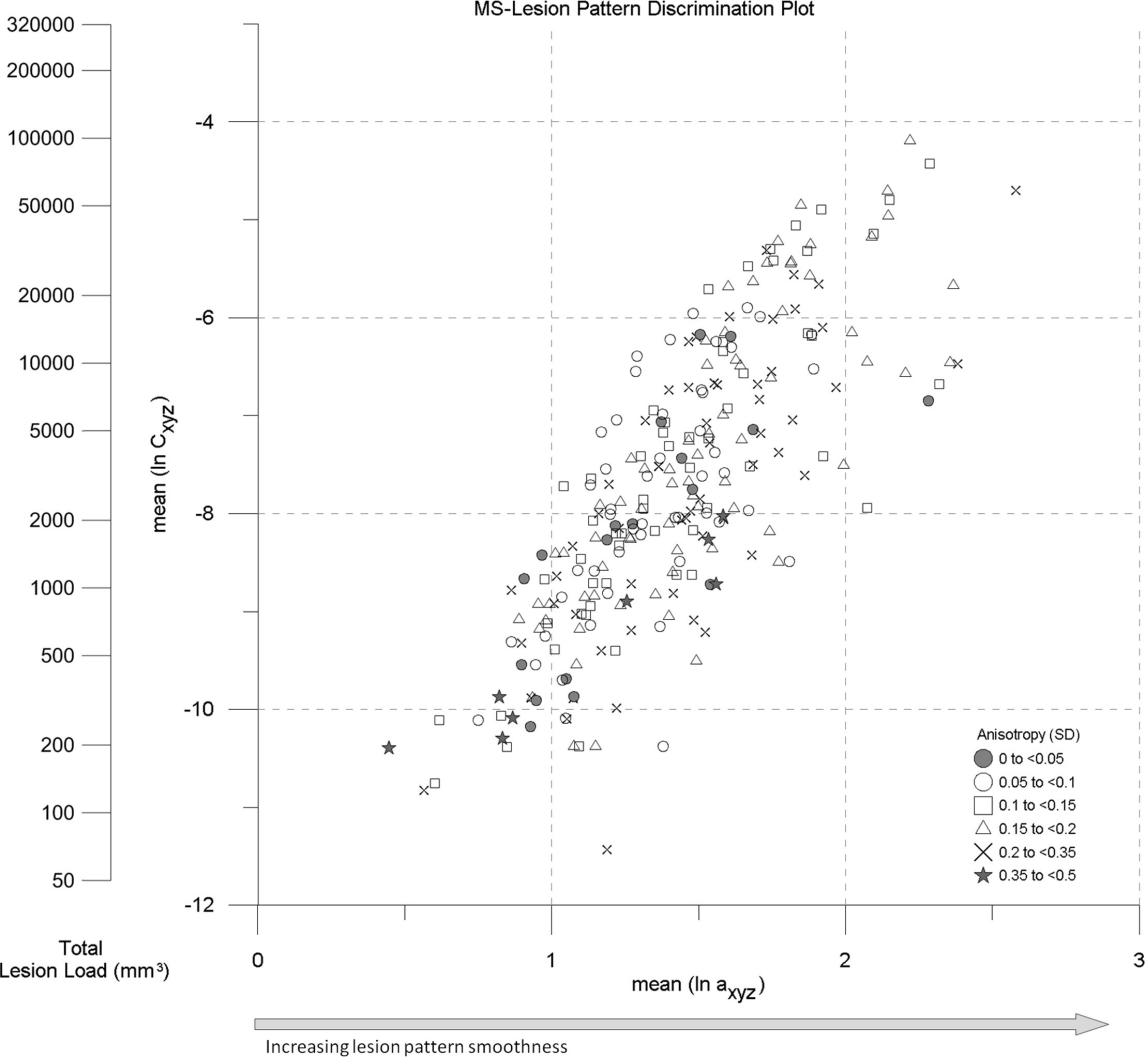
MS‐Lesion Pattern Discrimination Plot (LDP) combining the mean center (MC) positions and standard distance (SD) class symbols. Second *y* axis (nomogram) indicates total lesion load (TLL, mm^3^). Arrow indicates increasing pattern surface smoothness.

Combining the mean center and standard distance (SD) in a single plot, a compact representation of the spatial characteristics of MS‐lesion patterns is achieved. We term this plot the Lesion Pattern Discrimination Plot (Fig. 9, “LDP”). The LDP also indicates total lesion load, derived from the correlation in Figure 4(A).

Regarding spatial dispersion, the MS‐259 dataset shows isotropic and anisotropic patterns scattered over the point cloud except a concentration of extremely anisotropic patterns at very small total lesion loads which is attributed to aliasing in the representation of very small lesions by small numbers of voxels. Comparing the visualization of the MS‐259 dataset in the *a*‐*c* plot (Fig. 6) and in the LDP (Fig. 9), the LDP is more easily understood. The loss of information on *X*,* Y*,* Z* anisotropy indicated by individual symbols is counterbalanced by the introduction of standard distance symbols.

#### MS‐lesion pattern geometry and location in the LDP

**Figure 10:**
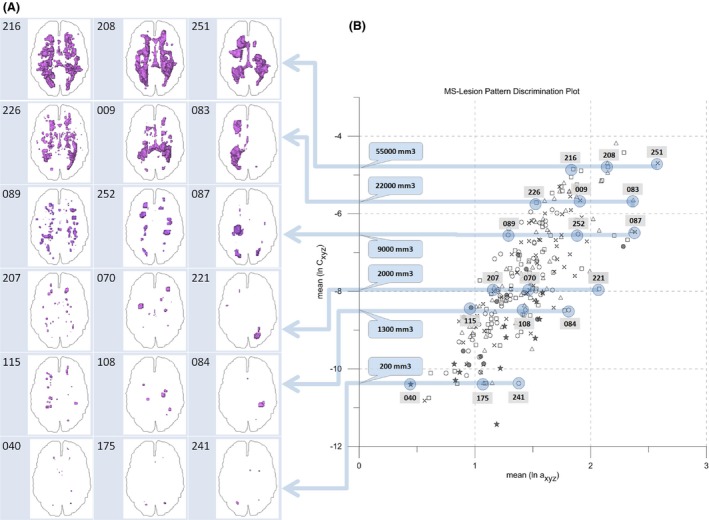
Comparison of projections of MS‐lesions to the axial plane with associated locations in the LDP. 18 MS‐lesion patterns are shown from the MS‐259 dataset. WM outline is shown for reference. See text for details.

Figure 10 is a synopsis of MS‐lesion pattern geometries (10a) and the corresponding positions in the LDP (10b). From the MS‐259 dataset, 18 patterns were selected that cover a large part of the populated area in the LDP. To ensure representativeness, six volume classes with a large spread (*a*_*max*_ *‐a*_*min*_) were chosen at iso‐volumes of 200, 1300, 2000, 9000, 22,000, 55,000 mm^3^. From each volume class three patterns were selected that represent the minimum, average, and maximum *a* (class volume ± 15%). The patterns and positions can be identified by numbers in Figure 10(A and B). Recalling the volume – surface considerations above, the LDP represents MS‐lesion pattern surface smoothness versus total lesion load. Working through Figure 10 reveals that complex patterns with many lesions or a “rough”/“complex” surface generally are positioned at the left fringe of the point cloud while patterns with few, big, and “smooth” lesions are placed toward the right border. Patterns around the long axis of the elliptic cloud mediate between rough and smooth extremes. This also holds quantitatively. For example, consider volume class 2000 mm^3^ in Table 1: proceeding from pattern wbles_207 via wbles_070 to wbles_221, the number of lesions decreases, surface area decreases, and volume per lesion, surface per lesion and the ratio of volume/surface area increases.

**Table 1:** Geometric characteristics of MS‐lesion pattern groups from the MS‐259 dataset. Groups represent similar total lesion load (“TLL”). See text for discussion

	ID	nLesions	Volume	Surface	Vol/Lesion	Surf/Lesion	Vol/Surf
Group	wbles_216	62	52,164	n.a.	841.35	n.a.	n.a.
	wbles_208	29	59,427	n.a.	2049.21	n.a.	n.a.
	wbles_251	8	52,373	23,645	6546.66	2955.63	2.21
Group	wbles_226	86	22,100	20,513	256.97	238.52	1.08
	wbles_009	33	23,031	15,922	697.91	482.48	1.45
	wbles_083	14	20,628	11,037	1473.43	788.36	1.87
Group	wbles_089	73	9470	10,389	129.73	142.32	0.91
	wbles_252	22	8657	6357	393.49	288.95	1.36
	wbles_087	12	8643	5142	720.28	428.50	1.68
Group	wbles_207	31	2187	2727	70.55	87.97	0.80
	wbles_070	14	2089	1867	149.22	133.36	1.12
	wbles_221	5	1941	1457	388.13	291.40	1.33
Group	wbles_115	23	1458	1987	63.39	86.39	0.73
	wbles_108	6	1293	1228	215.44	204.67	1.05
	wbles_084	4	1215	931	303.75	232.75	1.31
Group	wbles_040	13	219	403	16.88	31.00	0.54
	wbles_175	5	186	276	37.13	55.20	0.67
	wbles_241	4	172	245	43.03	61.25	0.70

Table 1 expresses the quantitative geometry behind Figure 10: groups are defined by their average total lesion volume (± 15%). Within each group the following holds: *a* increases top down and with increasing *a*, the number of lesions decreases, total surface area decreases, volume per lesion increases, surface per lesion increases, and the ratio of total volume/total surface area increases. In other words, at constant volume, with increasing *a*, pattern surface smoothness increases and surface complexity decreases.

#### Follow‐up examination expressed in the LDP

**Figure 11:**
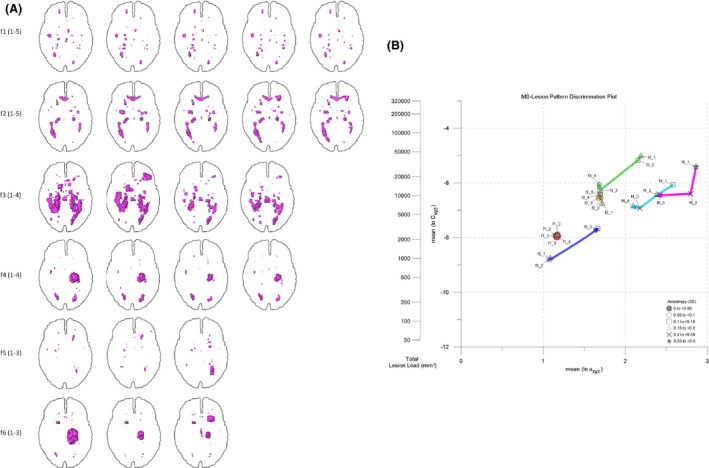
(A) Longitudinal studies f1_1–5_, f2_1–5_, f3_1–4_, f4_1–4_, f5_1–3_, f6_1–3_ (line‐oriented, order of follow ups from left to right). MS‐lesion patterns in projections to axial plane. See text for discussion. (B) LDP used to portray the evolution of MS‐lesion patterns f1_1–5_, f2_1–5_, f3_1–4_, f4_1–4_, f5_1–3_, f6_1–3_. Arrows indicate MS‐lesion pattern evolution paths. Color coding: f1 – red; f2 – gold; f3 – green; f4 – cyan; f5 – blue; f6 – magenta. See text for details.

The LDP is a versatile framework to portray lesion pattern evolution in follow‐up exams because it combines total volume, lesion pattern surface complexity and geometrical anisotropy information in a single, well‐arranged plot. Major changes as well as subtle fluctuations in MS‐lesion pattern geometry can be explored straightforwardly. As an example, the follow‐up exams of six MS‐cases (f1–f6) were documented in the LDP. The six cases differ with respect to lesion loads, lesion numbers, lesion pattern geometry, and lesion pattern evolution. Total investigation epochs range from 7 to 33 months and comprise three to five follow‐up exams of irregular duration. Figure 11(A) shows the follow‐up lesion patterns of cases f1,f2,f3,f4,f5,f6 in projections to the axial plane; Figure 11(B) gives the respective LDP entries (see also Table 2).

**Table 2:** Longitudinal data for cases f1_1–5_, f2_1–5_, f3_1–4_, f4_1–4_, f5_1–3_, f6_1–3_

ID	Date	Mean (ln(a))	Mean (ln(C))	SD
f1_1	2009‐02‐17	1.174	−7.920	0.164
f1_2	2009‐08‐10	1.147	−7.930	0.106
f1_3	2010‐02‐24	1.174	−7.880	0.118
f1_4	2011‐02‐08	1.163	−7.985	0.122
f1_5	2011‐11‐23	1.153	−7.940	0.185
f2_1	2009‐04‐22	1.718	−6.765	0.156
f2_2	2009‐12‐08	1.705	−6.546	0.096
f2_3	2010‐05‐10	1.674	−6.561	0.108
f2_4	2011‐06‐17	1.694	−6.434	0.089
f2_5	2011‐09‐26	1.695	−6.411	0.143
f3_1	2010‐09‐20	2.190	−5.024	0.148
f3_2	2010‐11‐02	2.153	−5.174	0.135
f3_3	2011‐02‐18	1.689	−6.288	0.123
f3_4	2011‐08‐12	1.681	−6.061	0.093
f4_1	2009‐10‐08	2.582	−6.076	0.122
f4_2	2009‐12‐21	2.384	−6.439	0.188
f4_3	2010‐08‐04	2.178	−6.930	0.205
f4_4	2011‐08‐11	2.096	−6.865	0.157
f5_1	2010‐02‐05	1.088	−8.761	0.180
f5_2	2010‐03‐24	1.072	−8.811	0.135
f5_3	2011‐09‐12	1.664	−7.673	0.135
f6_1	2011‐03‐02	2.862	−5.418	0.453
f6_2	2011‐04‐15	2.798	−6.403	0.274
f6_3	2011‐10‐20	2.401	−6.459	0.199

In the follow‐up examination of case f1, major geometric features remain constant, but minor fluctuations in smaller lesions show up (Fig. 11A); accordingly, in the LDP f1‐symbols plot closely together, but minor changes in anisotropy are indicated. Case f2 shows increasing total lesion load (TLL) over time, but the evolution path in the LDP indicates an approximately constant surface roughness: despite fluctuations, the evolution path runs approximately parallel to the volume axis. Case f3 has decreasing TLL from exams 1–3, but a small TLL increase in exam 4. The path connecting exams 1‐4 first runs towards the origin of the LDP but then points upwards in the last step. As TLL decreases, surface roughness increases due to the decomposition of large confluent lesion aggregates into smaller, mostly spherical ones. This is why lesion pattern anisotropy concurrently decreases. Case f4 is dominated by large, spherical lesions. A number of small elongated lesions accounts for a pronounced anisotropy. TLL decreases in all exams, but the last one. In the LDP, the pattern evolution path points towards the LDP origin except for step 4. Pattern surface roughness increases due to the decay of the large spherical lesions; there are major fluctuations in pattern anisotropy. Case f5 has a progressive trend with respect to TLL. Lesion pattern surface roughness decreases due to the confluence of several small lesions. The LDP evolution path points diagonally upwards. Case f6 shows decreasing TLL. Pattern surface complexity remains about constant between exams 1 and 2, but then increases due to concurrent formation of new lesions. In the LDP, the evolution path first runs approximately parallel with the ordinate and then takes a sharp bend to continue about perpendicular to increasing surface complexity at approximately equal TLL.

## Discussion

The geostatistical approach to MS‐lesion pattern characterization proposed and explored here is founded on the Theory of Regionalised Variables (ReV) in which a spatially continuous property is represented stochastically as a Random Function (RF). A RF is a stochastic generating mechanism which could have produced the data (represented as a random draw or ReV from the RF). Given second‐order stationarity (the parameters of the RF are spatially invariant), the variogram parameters, together with the mean, then characterize the RF, in particular capturing its spatial correlation properties. The MNI brain creates a Euclidean space which is then dissected into voxels of constant size spatially. The binary outcome of the MRI scanning process, expressed in this MNI space, is readily represented using the RF formalism, and the constant nature of its extent and support (voxel) from image‐to‐image facilitates excellent opportunities for sensitive comparison across members and through time. Indeed, in geostatistics, this situation is relatively rare. Therefore, we were able to interpret very small differences between images, and it was possible to place expectations, including minima and maxima, for each parameter estimated. For example, the MNI space also means that parameter values have clear interpretations in terms of volume and surface area relations.

The variogram represents a so called “two‐point statistic” in that the semivariance is calculated between two points (the present location and another at a given lag vector away; compare equation (1)). Two‐point statistics have only limited capabilities to describe the potentially complex spatial structures exhibited by MS‐lesion patterns. There is, thus, some trade‐off between the sensitivity afforded by application of the RF formalism to the standardized MNI space and the limited spatial representation afforded by the variogram. Moreover, empirical variograms of MS‐lesion patterns have to be limited to distances of 15 mm to enable meaningful variogram model fitting (Marschallinger et al. 2014). In making this restriction, some information on pattern granularity like repetitions (the so‐called hole effect) is lost. Moreover, the variogram is not sensitive to the absolute position of objects within a defined space.

Recently, much attention in geostatistics has been paid to multiple‐point geostatistics (MPG) (Strebelle 2000; Remy et al. 2009). The MPG formalism captures a much richer information set than can be obtained from two‐point statistics. For example, MPG has been used to represent properties such as hydraulic connectivity in sedimentary rocks, allowing modeling of properties such as permeability. Two‐point statistics are incapable of capturing and representing such properties. Thus, there is scope for exploration of the value of MPG for application to brain images.

While a natural choice given the fixed MNI space, the RF formalism is not the most natural interpretation of lesions. We tend to think of lesions as compact objects with fuzzy borders within the MNI space and this is particularly true in the representation afforded to us by the MRI scans in which lesions appear as having a more or less compact structure. This leads to alternative data models to the RF. The object‐based model has been applied widely in handling geographic information with great utility (Blaschke 2010), for example, in application to the classification of land cover in remotely sensed images. Recently, 3D object‐based image analysis applications have emerged in the biological and medical imaging domains (Schmidt et al. 2007; Marschallinger et al. 2011; Al Janab et al. 2012). The object‐based model has a lot to offer for the characterization of lesions, including the ability to handle each lesion separately, to logically link corresponding lesions in time‐series to track their status, and the ability to characterize the interrelations between lesions in a single image. Future research will focus on developing the MPG and object‐based models.

A further extension of our approach could be the inclusion of parameter uncertainty in the calculation of the mean geostatistical range and sill. By the use of simulations, this uncertainty could be used to identify significant changes in lesion volume and surface area relations between individual scans. This would represent a meaningful advantage of the modeling approach over the empirical analysis of those values.

## Conclusions

An efficient and computationally cheap geostatistics‐based method for characterizing MS‐lesion patterns from binarized and normalized MRI images was developed and presented. This approach enables the expression of key geometrical aspects of MS‐lesion patterns through estimation of the geostatistical range and sill (*a,c*) parameters which correlate with lesion pattern surface complexity and total lesion volume. The MS‐lesion pattern discrimination plot (“LDP”) introduced here and the *a*‐*c* plot are based on the above geostatistical parameters. The LDP communicates summary information on surface complexity, total volume and geometrical anisotropy of MS‐lesion patterns. The *a*‐*c* plot complements the LDP, informing on the preferred directional components of MS‐lesion patterns. The major advantage over existing methods is to achieve insight into the spatial development of whole MS‐lesion patterns (i.e., selective growth/decay in specific directions) without requiring object‐based/per‐lesion characterization. The approach also offers high precision and comparability between either different brains or the same brain at different times. Both the LDP and the *a*‐*c* plot are considered EDA tools adding to neurological standard image processing methods by quickly informing on the spatial or the spatiotemporal properties of MS‐lesion patterns in the course of cross‐sectional studies, longitudinal studies or the evaluation of medication efficacy.

## Conflict of Interest

None declared.
